# Evaluation of a *Stenotrophomonas maltophilia* bacteremia cluster in hematopoietic stem cell transplantation recipients using whole genome sequencing

**DOI:** 10.1186/s13756-017-0276-y

**Published:** 2017-11-13

**Authors:** Stefanie Kampmeier, Mike H. Pillukat, Aleksandra Pettke, Annelene Kossow, Evgeny A. Idelevich, Alexander Mellmann

**Affiliations:** 1 Institute of Hygiene, University Hospital Münster, Robert−Koch−Strasse 41, 48149 Münster, Germany; 2 Institute of Medical Microbiology, University Hospital Münster, Münster, Germany; 3 Science for Life Laboratory, Division of Translational Medicine and Chemical Biology, Department of Medical Biochemistry and Biophysics, Karolinska Institute, Stockholm, Sweden

**Keywords:** *Stenotrophomonas maltophilia*, Outbreak investigation, Whole genome sequence-based typing, Immunocompromised hosts

## Abstract

**Background:**

*Stenotrophomonas maltophilia* ubiquitously occurs in the hospital environment. This opportunistic pathogen can cause severe infections in immunocompromised hosts such as hematopoietic stem cell transplantation (HSCT) recipients. Between February and July 2016, a cluster of four patients on the HSCT unit suffered from *S. maltophilia* bloodstream infections (BSI).

**Methods:**

For epidemiological investigation we retrospectively identified the colonization status of patients admitted to the ward during this time period and performed environmental monitoring of shower heads, shower outlets, washbasins and toilets in patient rooms. We tested antibiotic susceptibility of detected *S. maltophilia* isolates. Environmental and blood culture samples were subjected to whole genome sequence (WGS)-based typing.

**Results:**

Of four patients with *S. maltophlilia* BSI, three were found to be colonized previously. In addition, retrospective investigations revealed two patients being colonized in anal swab samples but not infected. Environmental monitoring revealed one shower outlet contaminated with *S. maltophilia*. Antibiotic susceptibility testing of seven *S. maltophlia* strains resulted in two trimethoprim/sulfamethoxazole resistant and five susceptible isolates, however, not excluding an outbreak scenario. WGS-based typing did not result in any close genotypic relationship among the patients’ isolates. In contrast, one environmental isolate from a shower outlet was closely related to a single patient’s isolate.

**Conclusion:**

WGS-based typing successfully refuted an outbreak of *S. maltophilia* on a HSCT ward but uncoverd that sanitary installations can be an actual source of *S. maltophilia* transmissions.

## Background

*Stenotrophomonas maltophilia*, an intrinsically multidrug resistant gram negative pathogen, is widely distributed in aqueous habitats such as, sink drains, endoscopes and hemodialysis water within clinical settings [1, 2]. Usually, this pathogen is not highly virulent in immunocompetent individuals but can cause severe infections including bacteremia, peritonitis and meningitis in immunocompromised hosts resulting in complications, e.g. septic shock, respiratory failure, tissue necrosis and septic thrombophlebitis [3]. Recipients of hematopoietic stem cell transplantations (HSCT) are especially at risk and suffer from pulmonary hemorrhage resulting in higher mortality rates compared to non-HSCT patients [4].

In recent years, an increasing incidence of *S. maltophilia* is reported on oncologic wards [5]. Moreover, several outbreaks and pseudo-outbreaks caused by this pathogen have been reported [6, 7]. In addition to epidemiological investigations, different typing methods, e.g. pulse field gel electrophoresis (PFGE) and multi locus sequence typing (MLST) are used to identify the genetical relationship among different *S. maltophilia* isolates [8–11]. These techniques are useful in excluding outbreak scenarios, if MLST sequence types (ST) or PFGE patterns of isolated pathogens differ. In case of identical STs or highly similar PFGE patterns both methods reach their discriminatory limits. In these cases an outbreak is assumed, although more precise methods uncover and accidental cluster. Additionally, inter-laboratory comparability is limited using PFGE. Therefore, whole genome sequence-based typing (WGS) approaches are increasingly seen as gold standard method for highly discriminatory typing [12] and the superiority of WGS in comparison to other typing methods was already demonstrated for several bacterial pathogens [13–17]. Moreover, we could recently demonstrate the high reproducibility of WGS, which is another prerequisite for the applicability in clinical routine [18].

Here we evaluated a cluster of *S. maltophilia* bacteremia in recipients of allogeneic stem cell grafts using WGS-based typing.

## Methods

### Cluster detection, epidemiological investigations and infection control measures

**Fig. 1:**
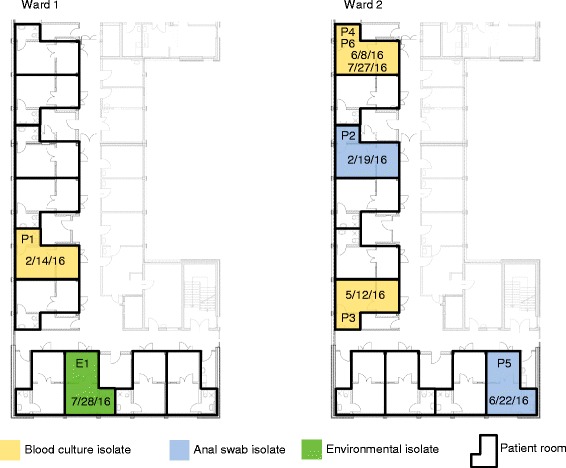
Distribution and isolation dates of *S. maltophilia* on the HSCT-unit. Distribution of blood culture (yellow), anal swab (blue) and environmental (green) isolates detected during February and July 2016 on both HSCT wards. Patient rooms on wards are highlighted by black edges. Dates of *S. maltophilia* detection are assigned to the according patient room

The 1500-bed University Hospital Münster includes two HSCT-units each comprising 10 patient rooms, which are all HEPA-filtered and equipped with separated bathrooms for each patient (Fig. 1). In total, 193 patients were admitted to the HSCT-units during 2016. Patients usually receive an allogenic HSCT.

*S. maltophilia* bloodstream infections (BSI) were defined as one or more blood cultures positive for *S. maltophilia* obtained from patients with clinical signs of infection (fever >38 °C, chills, hypotension) according to the critieria given by the European Centre for Disease Prevention and Control [19]. Four *S. maltophilia* BSI were detected in patients (P1, P3, P4, P6) in the HSCT ward between February and July 2016. As this number exceeds the average baseline of 0.5 *S. maltophilia* BSI per year detected during 2011–2015, an epidemiological investigation was initiated. Routine environmental screening of aqueous habitats (shower heads, shower outlets, washbasins and toilets) within patients’ rooms in the HSCT unit, normally focusing on multidrug resistant *Pseudomonas aeruginosa*, was expanded to *S. maltophilia.* Patients colonized with *S. maltophilia*, coincidentally detected in anal/rectal swabs or stool samples (as screening of HSCT-patients concentrates on multidrug resistant *Pseudomonas aeruginosa*), were retrospectively identified. Moreover, hand hygiene and surface disinfection measures were intensified.

### Identification and susceptibility testing

Positive blood culture samples were detected using an automated blood culture system (BACTEC™ 9240, Becton Dickinson GmbH, Heidelberg, Germany). Environmental samples were plated on Columbia sheep blood agar (Oxoid, Wesel, Germany) and MacConkey agar (Becton Dickinson, Heidelberg, Germany) after enrichment in Tryptic Soy Broth (Becton Dickinson) for 24 h at 36 °C. Subsequent species identification was performed by Matrix-Assisted Laser Desorption/Ionization-Time of Flight-Mass Spectrometry (MALDI-TOF MS; Bruker, Bremen, Germany). Susceptibility testing for trimethoprim-sulfamethoxazole (TMP-SMX) was performed using disk diffusion method in accordance with the European Committee on Antimicrobial Susceptibility Testing (EUCAST) standards and interpreted using EUCAST clinical breakpoints (version 6).

### Whole genome sequencing (WGS) based typing

To determine the clonal relationship of *S. maltophilia* strains isolated from blood cultures, the isolates were subjected to WGS using the Illumina MiSeq platform (Illumina Inc., San Diego, USA) as described previously [13]. Retrospectively identified colonization isolates were not subjected to WGS as they were no longer available in contrast to BSI isolates, that are stored for a longer time period. Using SeqSphere^+^ software version 2.0 beta (Ridom GmbH, Münster, Germany), all coding regions were extracted and compared in a gene-by-gene approach (core genome multilocus sequence typing, cgMLST) using *S. maltophilia* K279a strain (GenBank accession number AM743169.1) as a reference sequence. Instead of a published cgMLST scheme, which is not yet available, this ad hoc scheme was used to differentiate the cluster. SeqSphere^+^ software was used to display the clonal relationship in a minimum-spanning tree (MST). For backwards compatibility with classical molecular typing, i. e. MLST, the MLST sequence types (ST) were extracted from the WGS data in silico.

## Results

### Epidemiological investigation and susceptibility testing

Chronological order and spacial distribution of isolated *S. maltophilia* is displayed in Fig. 1. Isolates were obtained from blood culture samples of patients on both wards. Two of the four isolates (isolated from P4 and P6) were detected within short intervals in two patients both admitted to room 1 on ward 2. Of the four patients suffering from *S. maltophilia* bacteremia, three (P1, P4 and P6) were previously tested positive for *S. maltophilia* in stool samples or anal swabs. Additionally, two colonized patients (anal swabs; P2, P5) admitted to ward 2 were uncovered by the retrospective analysis (Table 1). One environmental isolate (E1) could be detected in a shower outlet of a patient room that was a patient with *S. maltophilia* infection. Susceptibility testing revealed all patient isolates to be susceptible to TMP-SMX, except isolate P3. The environmental isolate was resistant to TMP-SMX (Table 1).

**Table 1:** Patients with *S. maltophilia* colonizations and blood stream infections during February and July 2016

Patient no.	Anal colonization	Blood stream infection	Strain susceptibility to TMP-SMX	WGS performed
P1	+	+	S	+
P2	+	–	S	–
P3	–	+	R	+
P4	+	+	S	+
P5	+	–	S	–
P6	+	+	S	+

TMP-SMX – trimethoprim-sulfamethoxazole, WGS – whole genome sequencing

### Clinical characteristics and outcome of patients with *S. maltophilia* bacteremia

All patients with *S. maltophilia* BSI suffered from acute myeloid leukemia as the underlying disease. P2 and P5, who were only colonized by *S. maltophilia,* suffered from chronic lymphatic leukemia and myeloproliferative neoplasm. Except P1, all patients had already received allogenic SCT at the time of *S. maltophilia* detection. P1 and P6 developed an acute respiratory distress syndrome. P1, P3, and P6 died due to *S. maltophilia* bacteremia.

### WGS-based typing

**Fig. 2:**
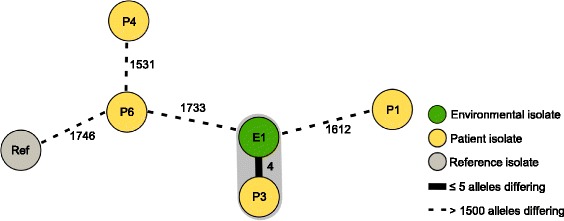
Minimum spanning tree of *S. maltophilia* isolates. Minimum spanning tree of four blood culture isolates (P, *yellow*), one environmental isolate (E, *green*) from the HSCT unit and one outgroup isolate (Ref, non-HSCT unit, isolated in May 2016, *grey*) based on up to 1876 target genes, pairwise ignoring missing values. Genotypes are consecutively numbered, starting with P1 (isolated in February 2016). Each dot represents one genotype and is colored according to its origin. Different connecting lines and numbers on these lines show the number of alleles differing between two genotypes

We analyzed four bloodstream isolates and the environmental isolate by WGS. Comparison via cgMLST, based on 1876 genes present in all isolates, revealed no genetical relationship among isolates originating from patients (Fig. 2). In contrast, *S. maltophilia* strains isolated from P3 and E1 showed a similar genotype with only four alleles difference, suggesting a relationship between these two isolates. P3 and E1 harbored the MLST ST 94. All other isolates harbored new MLST STs.

## Discussion

*S. maltophilia* has emerged as an important nosocomial pathogen associated with increased mortality rates in patients suffering from hematological malignancies and receiving hematopoietic stem cell transplantations [20, 21]. In this study, WGS was used to evaluate a cluster of four *S. maltophilia* BSI in patients partially suffering from pulmonary dysfunction associated with the infection due to this pathogen [4]. Susceptibility testing, normally used as a first indication of nosocomial transmission of bacterial strains, could not exclude a spread of one single clone. WGS and subsequent cgMLST analysis, methods that have shown to provide detailed information in evaluating the epidemiology in nosocomial clusters of other pathogens [22], excluded a genetical relation and therefore an outbreak scenario.

A number of different sources are possibly responsible for the increasing number of *S. maltophilia* BSI. Due to its multidrug resistant nature, selection of this pathogen can easily occur in patients after receiving broad-spectrum antibiotics [23]. Moreover, *S. maltophilia* infections occur frequently in combination with the distinct immunocompromised status of HSCT recipients [24]. On the other hand, several environmental sites such as sink drains could be detected as sources of *S. maltophilia* within hospital settings [25].

To what extent these habitats are origins for hospital-acquired colonizations or infections of *S. maltophilia* could not yet be shown in detail. In this study we documented a genetical relation between an environmental and a patient blood stream infection isolate by analyzing WGS data. Although there was no local proximity, *S. maltophilia* could be transmitted from the environment to a patient or vice versa, at least giving the possibility of further spread via aqueous habitats in the HSCT unit.

## Conclusion

WGS- analysis can be used to precisely refute an outbreak of *S. maltophilia*. Additionally, WGS- based typing documented sanitary installations in HSCT units to be an actual source or result of transmission between environmental and human habitats. Hence, in addition to classical infection control strategies, monitoring of aqueous habitats has to be established within the rooms of HSCT recipients in order to prevent transmission of these multidrug resistant organisms, as recently also shown for *P. aeruginosa* [26].

## Abbreviations

BSI: Blood stream infection
cgMLST: Core genome multilocus sequence typing
HSCT: Hematopoietic stem cell transplantation
MST: Minimum spanning tree
PFGE: Pulsed field gel electrophoresis
ST: Sequence type
WGS: Whole genome sequencing
